# Modulation of the Blood–Brain Barrier for Drug Delivery to Brain

**DOI:** 10.3390/pharmaceutics13122024

**Published:** 2021-11-27

**Authors:** Liang Han

**Affiliations:** Jiangsu Key Laboratory of Neuropsychiatric Diseases Research, College of Pharmaceutical Sciences, Soochow University, Suzhou 215123, China; hanliang@suda.edu.cn

**Keywords:** blood–brain barrier modulation, tight junction, active efflux, transcytosis, drug delivery

## Abstract

The blood–brain barrier (BBB) precisely controls brain microenvironment and neural activity by regulating substance transport into and out of the brain. However, it severely hinders drug entry into the brain, and the efficiency of various systemic therapies against brain diseases. Modulation of the BBB via opening tight junctions, inhibiting active efflux and/or enhancing transcytosis, possesses the potential to increase BBB permeability and improve intracranial drug concentrations and systemic therapeutic efficiency. Various strategies of BBB modulation have been reported and investigated preclinically and/or clinically. This review describes conventional and emerging BBB modulation strategies and related mechanisms, and safety issues according to BBB structures and functions, to try to give more promising directions for designing more reasonable preclinical and clinical studies.

## 1. Introduction

**Figure 1:**
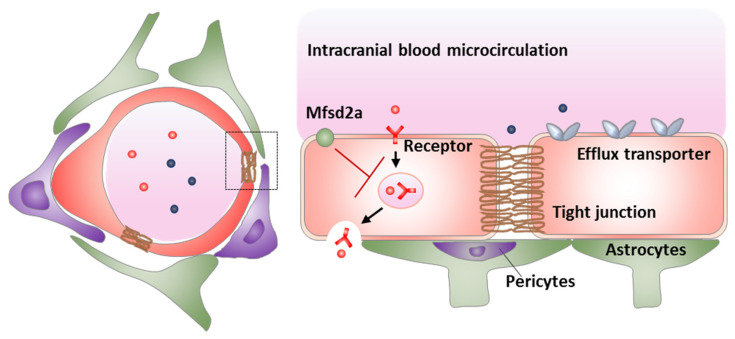
The neurovascular unit (**left**) and the mechanisms of transport inhibition by the BBB (**right**).

The blood–brain barrier (BBB) plays a crucial protective role in maintaining a highly precise brain microenvironment for neuronal activity by regulating material transport into and out of the brain. The structural bases of the BBB (Figure 1) are brain capillary endothelial cells with tight junctions, active efflux transporters, and major facilitator superfamily domain-containing protein 2a (Mfsd2a), which jointly endow the BBB with extremely low both paracellular permeability and transcellular permeability [1]. Tight junctions seal endothelial paracellular gaps, leading to high trans-endothelial electrical resistance and limited paracellular transport. Transmembrane tight junction proteins include claudins, occludin, and junctional adhesion molecules, which all attach to intracellular actin cytoskeleton by membrane-associated proteins (e.g., zonula occludins-1). Highly expressed active efflux transporters include P-glycoprotein (Pgp), breast cancer resistant protein (BCRP), and multidrug-resistance proteins (MRPs). Mfsd2a mediates unique BBB endothelial lipid composition via transporting lysophosphatidylcholine esterified docosahexaenoic acid to BBB endothelial cells, to limit formation of caveolae-mediated transcytotic vesicles [2,3,4]. In addition, endothelial cells, pericytes, and astrocytes jointly form the neurovascular unit (Figure 1), and regulate the development and function of the BBB microcirculation by interacting with each other via secreting several factors [5,6,7]. These above properties cause the BBB to be constantly and dynamically modulated by both physiological and pathological factors [8,9].

Despite its protective function, the BBB blocks the entry of therapeutic substances into the brain. Although various brain diseases can lead to BBB breakdown with impaired structure and increased permeability [8], BBB around lesion margins or after repairing (e.g., Pgp upregulation in epilepsy and brain tumor) can still block drug delivery to the brain [9,10,11,12]. Therefore, systemic drug therapy for brain diseases is severely limited by the BBB. BBB modulation contributes to an increased drug concentration in the brain, and thus increases the efficiency of various systemic therapies [13]. Crucial proteins and structures in formation and regulation of BBB and their changes in brain diseases have been selectively regulated to improve drug delivery for systemic therapies against various brain diseases.

This review describes various conventional and emerging strategies for BBB modulation that increase both paracellular permeability and transcellular permeability of the BBB, and classifies these strategies according to BBB structures and functions including tight junctions, active efflux, and low transcytosis (Table 1). Furthermore, mechanisms responsible for increased BBB permeability and safe issues related to various strategies are also discussed, to try to give more promising directions for designing more reasonable preclinical and clinical studies.

## 2. Modulation of Tight Junctions

**Figure 2:**
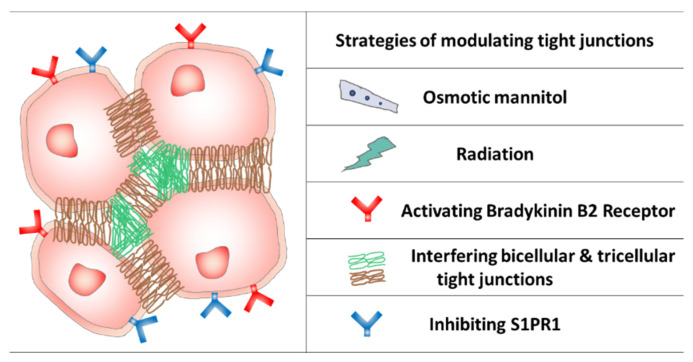
The BBB tight junctions and typical modulation strategies.

Opening BBB tight junctions is supposed to increase paracellular BBB permeability and facilitate paracellular drug transport into the brain [14]. Ideally, tight junction opening should be transient and selective in a controlled manner to prevent unwanted accumulation (and toxicity) in the brain, and also avoid any short- or long-term peripheral side effects [15]. Various tight junction opening strategies have been reported with robust both preclinical and clinical performance (Figure 2). However, concerns of causing severe toxicity constantly exist, because the non-specific accumulation of neurotoxic blood components may induce neuronal degenerative changes and even cognitive impairments [16,17,18]. Various reported strategies are discussed here, which may help to promote the emergence of highly efficient approaches with minimal side effects.

### 2.1. Osmotic BBB Disruption

Intra-arterial infusion of 25% hyperosmotic mannitol into the carotid or vertebral artery can induce vasodilation, endothelial cell shrinkage, and subsequent tight junction loosening and separation, leading to transient and reversible BBB disruption [15,19]. While conventional intra-arterial administration increases drug exposure of brain tumors 10-fold, osmotic BBB disruption can further increase drug exposure by up to 100-fold [20]. This strategy has been translated into the clinic to increase chemotherapy efficiency for brain tumors, and the tight junction opening window by osmotic BBB disruption can last for hours in humans [21]. Other hyperosmotic agents that transiently open tight junctions also include arabinose, lactamide, saline, urea, and radiographic contrast agents [15]. Osmotic BBB disruption is generally nonselective with uncontrolled flow into whole brain regions, such as neurotoxic blood components (e.g., albumin), leading to edema, neurological toxicity, epilepsy, aphasia, and hemiparesis [15,22,23,24]. In addition, the invasive nature and general anesthesia render the technique impractical for drug therapy against chronic brain diseases [14]. Therefore, the use of osmotic BBB disruption is confined to only clinical management of brain tumors.

### 2.2. Radiation-Mediated BBB Disruption

Radiation cannot only induce tumor cell apoptosis, but also disrupt the BBB [7,18,25,26,27,28,29,30,31]. Although the underlying mechanisms are still uncertain, BBB disruption induced by radiation leads to enhanced paracellular diffusion and transcellular transport [7]. Radiation therapy has been combined with systemic therapies to treat brain tumors. Although some study suggests that radiation failed to increase intracranial drug concentrations, increased gefitinib concentration in cerebrospinal fluid was shown with escalating radiation dose in patients with brain metastases in clinical trials [32,33]. Therefore, further research is needed to verify whether enhanced drug delivery to the brain can indeed occur after radiation and whether it is based on the effects on the BBB [34]. It has been reported that the disrupted BBB by radiation needs hours to years to recover [27]. Therefore, irradiation involves acute, subacute, and chronic dose-dependent toxicity [26,27]. For example, vasogenic edema from vascular damage causes early radiation toxicity syndrome including headache, nausea, or neurologic deficits [18]. Subacute side effects may appear around six months post radiation and progress into chronic dysfunction. Chronic side effects include radiation-induced necrosis, demyelination, leukoencephalopathy, cerebral atrophy, and neurocognitive deficits, and so on [35,36]. Stereotaxic radiosurgery may be an alternative approach to reduce radiation-related intracranial side effects and simultaneously maintain the BBB disrupting effects.

### 2.3. Activating Bradykinin B2 Receptor

Bradykinin B2 receptor is constitutively expressed on BBB endothelial cells. Its stimulation can rapidly and transiently disengage tight junctions and increase BBB permeability [37]. The expression of bradykinin B2 receptor is upregulated in the blood–tumor barrier (BTB) in brain tumors [38,39]. Therefore, activating the bradykinin B2 receptor may selectively modulate the BTB permeability and increase drug delivery to brain tumors. This strategy may be able to avoid side effects of osmotic BBB disruption towards the normal brain, owing to targeting effects on the BTB. Nonapeptide RMP-7 can selectively stimulate bradykinin B2 receptor and possesses longer blood circulation than endogenous bradykinin [37]. RMP-7 has been shown to be effective in opening BBB tight junctions and increasing intracranial drug concentrations in normal animal and in brain tumor animal models after intravenous infusion or intra-arterial injection [40,41,42].

Bradykinin B2 receptor is also expressed at numerous additional sites, and its activation at these sites can induce a wide variety of physiological responses including smooth muscle relaxation (e.g., vasculature) and contraction (e.g., intestine and uterus), inflammation modulation, pain mediation, and dose-limiting side effects (e.g., hypotension) [37]. The major side effects of intravenously administered tolerable RMP (up to 300 ng/kg over 10 min) were immediate and transient and included flushing, nausea, headache, and tachycardia [43,44,45]. At clinically approved dosage, the effects of intravenously infused RMP-7 weren’t shown in Phase II clinical trials in patients with brain tumors [38,44,45,46]. Intracarotid injection rather intravenous infusion has the potential of concentrating RMP-7 to the brain and reducing effects on peripheral tissues. Except for transient decreases in arterial blood pressure, intra-arterial administration of RMP-7 wasn’t shown to produce any other side effects, such as apparent cerebrovascular abnormalities and neurologic deficits in swine [47]. It is to be noted that bradykinin-increased BBB permeability may also be related with increased vesicular transport [48]. Considering the specific effect of RMP-7 on the BTB and the evidence demonstrated with the U87 glioma model that 7~100 nm pores in BTB are sufficient to allow the translocation of certain nanoparticles [49], the possibility of combining RMP-7 with targeting macromolecules or nanomedicine should be further evaluated.

### 2.4. Direct Interference of Tight Junctions

Claudins are major components of tight junctions, and claudin-5 dominates the BBB tight junctions by limiting paracellular penetration of small molecules [50,51,52]. Knockdown of BBB endothelial claudin-5 using specific siRNA was also shown to be able to transiently and reversibly increase BBB permeability to small molecules (MW up to 742) in mice [53]. The BBB opening and increased permeability after claudin-5 siRNA treatment were found to be size-selective and last for 72 h for small molecules with MW 443 and for 48 h for small molecules with MWs 562 and 742. It is also noteworthy that BBB opening after claudin-5 siRNA treatment also contributed to the clearance of water from the brain with cognitive improvement in mice with focal cerebral edema [54]. Anti-claudin-5 antibody can specifically recognize and bind with the extracellular loop domain of claudin-5, leading to impaired BBB tight junctions and increased BBB permeability to small molecules (e.g., sodium fluorescein with MW 376) [55,56,57]. The 3 mg/kg antibody didn’t induce any liver and kidney injury, change of plasma biomarkers of inflammation, and behavior change in cynomolgus monkeys while vasodilation in liver, lung, and kidney, lung hemorrhage, and brain edema were shown with 6 mg/kg antibody [55]. The side effects of high dose of anti-claudin-5 antibody can be ascribed to the wide expression of claudin-5 in the vascular endothelium of peripheral tissues [52]. The narrow window between the tight junction opening and peripheral side effects should be considered and local delivery of anti-claudin-5 antibody may be able to prevent the above side effects. Peptide C5C2 can bind with the first extracellular loop of claudin-5 and was shown to internalize and downregulate claudin-5 [58]. However, in contrary to anti-claudin-5 antibody and claudin-5 siRNA, the transient and reversible BBB opening mediated by C5C2 was found to allow brain entry of molecules up to 40 kDa.

Angulin-1 and tricellulin constitute the functional BBB tricellular tight junctions, which blocking brain entry of macromolecules only [50,59,60]. Angubindin-1 is derived from the receptor-binding domain of *Clostridium perfringens* iota-toxin and can bind with angulin-1 of tricellular tight junctions and remove angulin-1 and tricellulin from tricellular tight junctions, leading to enhanced BBB permeability to macromolecules [61]. Intravenously injected angubindin-1 disrupted tricellular tight junctions without any overt adverse effect and increased BBB permeability for transient brain entry of macromolecules [60].

### 2.5. Other Potential Strategies

There also reported numerous other strategies for opening BBB tight junctions with enormous potential. For example, as a G protein-coupled receptor, sphingosine 1-phosphate receptor-1 (S1PR1) plays an important role in the barrier function of the BBB and peripheral vessels [17]. Knockout or downregulation of endothelial S1PR1 transiently and reversibly altered distribution of BBB tight junction proteins and allowed increased brain penetration of small molecules with MW less than 3000 in mice. The opening of BBB tight junctions by S1PR1 inhibition via FTY720 didn’t show any signs of brain inflammation or injury. Controversially, FTY720 was also reported to reverse downregulation of S1P1 and S1P3 in retinas of diabetic rats and repair BBB by upregulating claudin-5 and downregulating VCAM-1 [62,63]. Therefore, further research is needed to verify whether FTY720 can indeed open BBB tight junctions and enhance paracellular drug delivery to the brain. The upregulation of astrocytic S1PR3 was linked to high permeability of brain metastases from breast cancer [64,65,66], suggesting contrary pathophysiological effects of S1PR3 to those of S1PR1. Further studies are also needed to elucidate the respective roles of S1PR1 and S1PR3 in the BBB.

Intracarotid injection of alkylglycerols was shown to transiently increase paracellular BBB permeability to small molecules and macromolecules with an efficiency comparable to that of osmotic BBB disruption and higher than that of intracarotid infusion of bradykinin [67,68,69,70,71]. Although intracarotid administration is an invasive procedure and the effects of alkylglycerols haven’t been proven clinically, the strategy of alkylglycerol-mediated BBB opening didn’t reveal any sign of toxicity at the animal level after long-term in vivo analyses [71]. In addition, intracarotid infusion of oleic acid or linoleic acid was also found to cause reversible BBB disruption and increase BBB permeability, but with brain edema, necrosis, and demyelination [72,73].

In theory, selectively disrupting the diseased BBB is more advantageous than nonspecific BBB disruption when systemic therapy of brain diseases is considered, owing to the absence of unwanted side effects to normal brain regions, e.g., the strategy of activating the bradykinin B2 receptor in 2.3. Pericytes derived from glioblastoma were reported to be directly associated with the BTB tight junctions and poor response of glioblastoma to chemotherapy [74]. Reducing pericyte coverage of the BTB was found to successfully increase paracellular BTB permeability and then improve chemotherapy efficiency against glioblastoma [75]. Ibrutinib with the ability of eliminating glioblastoma-derived pericytes by inhibiting BMX kinase was proven to be able to selectively impair the BTB tight junctions to enhance the therapeutic efficacy of drugs with poor BTB penetration [74].

Substance P is an important proinflammatory neuropeptide that functions as an immunoneuromodulator in the brain. Notably, substance P is also expressed by breast cancer and involves in chemoresistance and BBB crossing of breast cancer cells to form brain metastases [76]. Substance P secreted by breast cancer cells induces BBB endothelial cells to successively secret tumor necrosis factor alpha (TNFα) and angiopoietin-2 (Ang-2), which further activate their receptors to reorganize endothelial cytoskeleton and destabilize inter-endothelial adhesion complexes to alter distribution of tight junction proteins such as claudin-5 [76,77,78,79]. In addition, increased BBB permeability by secreted Ang-2 is also correlated with upregulated caveolin-1 and intensified caveolae-mediated vesicular transport [80]. Considering the substance P-mediated effects and corresponding specific expression of TNF receptors in the BTB (brain metastases), substance P, TNFα, Ang-2 and their derivatives can be used to open tight junctions in the BBB and tumor-associated BTB, respectively.

## 3. Modulation of Active Efflux

Active efflux transporters are selective gatekeepers at the BBB and cooperate with tight junctions to regulate substance transport into and out of the brain. Pathophysiological processes and pharmacological intervention further aggravate the efflux effect by intensifying expression and activity of these efflux transporters. Therefore, targeting regulatory pathways of BBB efflux transporters is supposed to be a feasible approach for efficient drug delivery to the brain [81,82]. BBB efflux transporters include Pgp, BCRP and MRPs. Although the role of other efflux transporters may be underestimated, Pgp with multiple binding domains for broad substrate spectrum is thought to be a predominant BBB efflux transporter [81,82]. Therefore, this section is focused on the modulation of Pgp. Typical strategies including direct inhibition and inhibiting transcriptional activation are introduced here. Notably, preserving and restoring their normal expression and activity after treatment is of specific importance, owing to the protective roles of active efflux. Many other modulating mechanisms of BBB Pgp expression and activity, such as posttranscriptional mechanisms, posttranslational mechanisms, and intracellular and intercellular trafficking, were not reviewed here, owing to the absence of reported pharmacological intervention [81].

### 3.1. Direct Inhibition of Efflux Transporters

**Figure 3:**
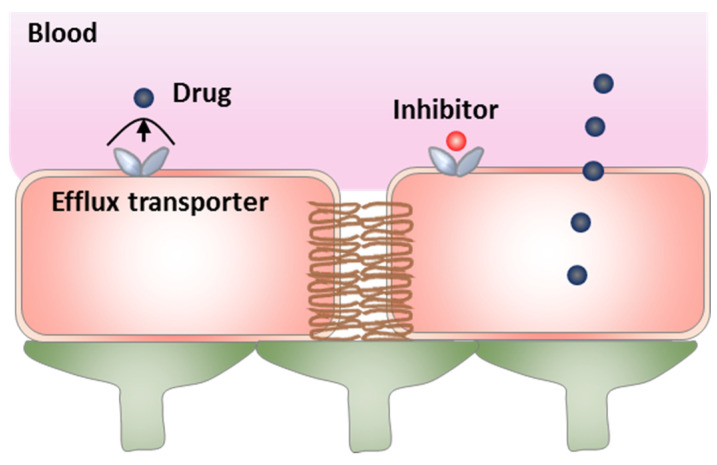
The strategy of directly inhibiting efflux transporters.

Pgp activity can be directly inhibited using specific competitive inhibitors, such as verapamil (Figure 3) [83,84]. In vivo cerebral microdialysis can be used to directly measure the concentration of free drug in the brain to study possible drug–Pgp interactions [85]. For example, through brain microdialysis in rats, it has been shown that Pgp inhibition enhanced the brain concentration of Pgp substrates ceftriaxone and seliciclib [86,87]. Evaluated by intracerebral microdialysis on mice, topotecan penetration in gliomas was enhanced by modulating Pgp using gefitinib [88]. However, high dosed inhibitors are often used, owing to their low Pgp binding affinity and greater resistant Pgp inhibition at the BBB than peripheral Pgp [89], which may lead to tolerability concerns and side effects. In addition, Pgp inhibition at the BBB can enhance brain concentrations of unwanted substrates, which could lead to serious intracranial side effects from the unwanted compounds [85]. The second-generation inhibitors with improved tolerability, including valspodar, possess the shortcomings of inhibiting cytochrome P450 enzymes, leading to delayed drug clearance and prolonged systemic exposure of co-administered therapeutic drugs [82]. Thus, the effects on drug metabolism and pharmacodynamics limit the application of these two generation inhibitors. Third-generation inhibitors (e.g., elacridar) affect BBB efflux efficacy by inducing Pgp conformation changes.

### 3.2. Targeting Regulatory Pathways of Efflux Transporters

Inhibiting the signal pathways intensifying Pgp expression and activity is supposed to overcome Pgp-mediated efflux and chemoresistance [90]. A number of “orphan” nuclear receptors are key transcriptional regulators and their expression at the BBB can upregulate Pgp, BCRP, and MRPs to respond to potentially harmful compounds. For example, pregnane X receptor (PXR) directly participates in Pgp upregulation by anticancer drugs [91,92,93]. Antagonists of these orphan nuclear receptors, such as ketoconazole, were shown to effectively inhibit rifampicin- and paclitaxel-induced Pgp upregulation, and sensitize brain cancers to anticancer drugs [94]. It is to be noted that these Pgp regulating mechanisms at the BBB likely exist in peripheral tissues. Strategies reversing Pgp upregulation might also reduce Pgp in other tissues and thereby cause unintended side effects.

**Figure 4:**
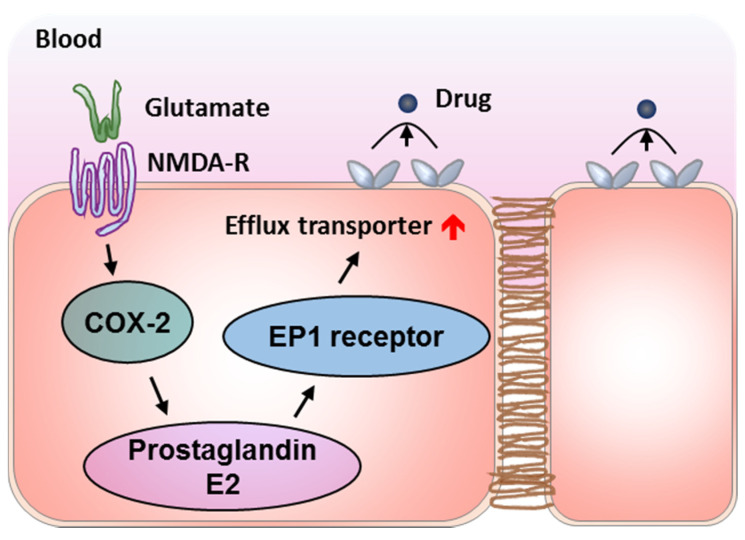
The signaling pathway of glutamate/NMDA-R/COX-2/prostaglandin E2 EP1 receptor induces the upregulation of efflux transports in epilepsy.

The signaling pathway of glutamate/NMDA-R/COX-2/prostaglandin E2 EP1 receptor induces Pgp and BCRP overexpression at the BBB in epileptic brains (Figure 4). MK-801, an antagonist of N-methyl-D-aspartate receptor (NMDA-R), was proven to effectively prevent glutamate-induced Pgp upregulation [95]. However, the side effects severely restrict the development of this approach [96]. COX inhibition using indomethacin and celecoxib was proven to prevent seizure-induced Pgp overexpression and enhance delivery of antiepileptic drugs to the brain in epilepsy model with negligible effect on basal Pgp expression and transport activity [97,98,99]. Unfortunately, COX-2 inhibitors are also associated with an enhanced risk of cardiovascular and cerebrovascular events and the controversial impact on seizure thresholds and seizure severity [100]. Inhibiting the prostaglandin E2 EP1 receptor by SC-51089 was further demonstrated to abolish glutamate-induced Pgp increases at the BBB, and enhance antiepileptic drug efficacy [82,101]. Neurodegeneration aggravation after COX-2 inhibition can be attributed to the blocking of EP2, EP3, and EP4 downstream of prostaglandin E2 [102,103,104]. Therefore, antagonism of the prostaglandin E2 EP1 receptor may be the most promising approach to control Pgp expression and enhance entry of antiepileptic drugs to epileptic brains. Strategies of reversing Pgp upregulation in epilepsy can be extended to the application in treating brain ischemia, because the glutamate release and similar Pgp upregulation mechanisms also exists in brain ischemia [105]. In contrary, as a critical factor for intracranial clearance of amyloid β-protein (Aβ), Pgp expression at the BBB is often downregulated to promote intracranial Aβ accumulation in Alzheimer’s disease [106,107,108,109]. Signaling pathways inducing Pgp upregulation may be carefully harnessed to treat Alzheimer’s disease. For example, PXR ligands (e.g., hyperforin) and EP1 receptor agonists hold the potential for upregulating Pgp to interfere with Alzheimer’s disease. In addition, strengthening the Wnt/β-catenin signaling may also be able to increase Pgp to reduce Aβ burden in Alzheimer’s disease [110].

## 4. Modulation of Transcytosis

Receptor-mediated transcytosis is often used to mediate transcellular BBB crossing, owing to the extremely low paracellular BBB permeability controlled by the tight junctions and active efflux transporters. Receptor-specific ligands can be used to decorate drug delivery systems (e.g., multifunctional nanoparticles) to initiate transcellular transport across the BBB [8,49,111,112,113,114,115,116,117]. However, the density of these target receptors at the BBB is much lower than that of nutrient transporters (e.g., glucose transporter) [118]. More importantly, exclusively expressed Mfsd2a limits formation of caveolae-mediated transcytotic vesicles and the transcytosis rate at the BBB by regulating BBB endothelial lipid composition [1,2,3,4,5,6,119,120,121]. Therefore, the efficiency of transcellular transport at the BBB should be modulated to improve brain accumulation of ligand-modified drug delivery systems.

### 4.1. Upregulation of LRP1

Low-density lipoprotein receptor-related protein 1 (LRP1) is expressed at both luminal and abluminal sides of the BBB. While abluminal LRP1 is primarily responsible for clearing Aβ from the brain to blood [122], luminal LRP1 has been extensively studied to mediate drug delivery to the brain. Inspired by the fact that statins can suppress cholesterol synthesis and then induce compensatory expression of LRP1 [118,123,124,125,126], simvastatin-loaded nanoparticles were reported in our previous work to upregulate LRP1 at the BBB and boost LRP1-targeting chemotherapy efficiency against brain metastases from breast cancer [114]. In addition, LRP1 can respond to astrocytic apolipoprotein E to maintain the BBB integrity by suppressing the BBB-degrading pathway of activation of cyclophilin A-matrix metalloproteinase 9 [127,128]. More importantly, the diminishment of abluminal LRP1 is closely related to intracranial Aβ accumulation in Alzheimer’s disease, and also to the aggregation of α-synuclein into Lewy bodies in Parkinson’s disease [127,128]. Therefore, the strategy of upregulating LRP1, a potentially important therapeutic target of BBB breakdown-related diseases, holds the potential to be used to treat both Alzheimer’s disease and Parkinson’s disease. In fact, delivery of LRP1 gene to the BBB has been reported to facilitate Aβ clearance via upregulating LRP1 [127,129].

### 4.2. Inhibition of Mfsd2a

Reversible inhibition of Mfsd2a holds the potential to temporarily liberate the limited transcytosis at the BBB [2]. In our previous work, Mfsd2a inhibitor tunicamycin was delivered to the BBB and shown to be able to enhance brain accumulation of subsequent therapeutic nanoparticles and the efficiency in treating brain metastases from breast cancer in mice [117]. Owing to the crucial role of Mfsd2a in transporting essential fatty acids and promoting BBB formation and brain development, Mfsd2a knockout induces microcephaly, Allan-Herndon-Dudley syndrome, and other severe side effects (e.g., BBB breakdown, neuronal loss, cognitive impairment, intellectual disability, behavioral deficits, spasticity, and absent speech and so on) [4,121,127,128,130,131,132]. In clinical practice, the loss of BBB Mfsd2a is often found in Alzheimer’s disease, traumatic brain injury, stroke, and brain tumor. Mfsd2a may be a potential therapeutic target for these diseases and remains to be explored further [130,131]. However, tunicamycin-mediated Mfsd2a inhibition is likely to be reversible, because the inhibition mechanism is supposed to be just physical binding and the inhibitor would dissociate from Mfsd2a after entering the brain [2]. Therefore, side effects associated with Mfsd2a deficiency could be avoided.

### 4.3. Upregulation of GLUT1

Glucose transporter 1 (GLUT1) at the BBB maintains the continuous high glucose and energy demands of the brain. Based on its essential role in transporting glucose and its participation in pathological processes of various brain diseases such as Alzheimer’s disease, ischemia, and brain tumors, upregulation of GLUT1 has been proposed to treat hypoglycemic conditions, while its downregulation or inhibition could be used to cope with hyperglycemic conditions [133]. In addition to being direct therapeutic targets, wide expression of GLUT1 at the BBB has been extensively used to mediate drug delivery to the brain. GLUT1 upregulation at the luminal side of the BBB via hypoglycemia and its migration to the abluminal side were implemented via rapid glycemic increase after fasting [134]. Then, the brain accumulation of properly configured glucose nanoparticles was shown to reach 6% dose/g-brain in normal mice with glycemic control. Because Alzheimer’s disease is characterized by reduced GLUT1 at the BBB and a reduction of glucose transport [129], this strategy of rapid glycemic increase after fasting holds the potential to treat Alzheimer’s disease via upregulating GLUT1.

## 5. Multifunctional Strategies by Multiple BBB Modulation

All the above strategies increase BBB permeability via separately modulating tight junctions, active efflux, or transcytosis. In fact, there many other multifunctional strategies were also reported, which can simultaneously modulate multiple controlling factors and achieve theoretically higher BBB permeability for efficient drug delivery to the brain.

### 5.1. Focused Ultrasound

Low intensity focused ultrasound is a noninvasive technique that is combined with intravenously injected gaseous perfluorocarbon-filled microbubbles to transiently and focally modulate the BBB [135,136]. With the help of stable oscillation of microbubbles, the BBB is transiently and reversibly disrupted and characterized by (1) disintegration of tight junctions including claudin-5; (2) fenestration and channel formation; (3) Pgp suppression; and (4) upregulation of caveolin-1 and caveolae-mediated transcytotic vesicles, which jointly facilitate both paracellular transport and transcellular passage through the BBB [137,138,139,140,141]. Under the guidance by magnetic resonance imaging, microbubble-enhanced focused ultrasound can act on specific intracranial areas with negligible toxicity to adjacent normal brain cells [142,143,144,145,146]. Further, ultrasmall superparamagnetic iron oxide nanoparticles can be encapsulated into microbubbles and nanobubbles to increase the BBB disruption efficiency and monitor post-sonication BBB opening and drug delivery across the BBB [147,148]. Generally, microbubble-enhanced focused ultrasound is less invasive than BBB disruption induced by osmotic agents with minimal neurotoxicity, inflammation, and stroke occurrences in clinical settings [135,149,150,151,152,153]. However, increasing acoustic energy is associated with increasing risk of side effects including vascular damage, edema, parenchymal damage, microhemorrhage, and over-activation of the immune system (e.g., autoimmunity) [137,154,155]. Therefore, adjusting ultrasound parameters is necessary for reducing risks, especially for repeated treatments and the application of mediating drug delivery to Alzheimer’s disease owing to Aβ-mediated resistance of BBB disruption [156,157].

### 5.2. Activating A2A Adenosine Receptor

A2A adenosine receptor interacts with G_s_ protein to activate adenylate cyclase and further increase intracellular cAMP [154]. It is located on platelets, leukocytes, blood vessels and intracranial regions such as striatum [158]. Its activation can inhibit platelet aggregation and regulate blood pressure through vasodilation [159]. Its expression can be altered by pathological conditions, e.g., upregulation on glial cells by hypoxia and inflammation and at the BBB by brain tumors [10,160], to protect against damage via reducing inflammation [161]. The activation of A2A adenosine receptor at the BBB can increase BBB permeability by rapid and reversible decrease of tight junction proteins (e.g., claudin-5), Pgp and BCRP [154,162]. Intravenous injection of clinically used regadenoson was shown to be able to increase intracranial concentrations of small molecules and macromolecules in preclinical studies [163,164,165,166,167]. However, regadenoson treatment at FDA-approved doses in humans (bolus injection of 0.4 mg) was found without increased intracranial concentrations of temozolomide in patients with recurrent glioblastoma [168,169], which may be attributed to the insufficient dose of this strategy for effective BBB modulation, and warrants the necessity of studies on whether higher dose or different agonists would be effective [154]. The alternative option of nanomedicine-mediated targeted agonist delivery holds the potential of not only enhancing selectivity, intensifying the BBB opening effect, and prolonging the BBB opening time window from up to 50 min to up to 2 h [170,171,172,173,174], but also avoiding affecting peripheral A2A adenosine receptors to minimize systemic side effects, e.g., excessive vasodilatation of the peripheral vascular bed, dizziness, and headaches [154].

### 5.3. Activating Potassium Channels

Blood vessel endothelium widely expresses potassium channels, especially ATP-dependent potassium channels (*K*_ATP_) [175,176]. Activation of *K*_ATP_ can regulate vascular hyperpolarization, relaxation, dilatation and vessel permeability [175,176,177,178], making *K*_ATP_ a therapeutic target for hypertension. *K*_ATP_ is often upregulated in hypoxic environments including brain tumors and ischemia [178,179]. The regulatory effects on BTB permeability by activating the *K*_ATP_ are expected to be more significant than those of the BBB [176,180]. These effects include intensified paracellular diffusion and transcellular transport, which involve in downregulated tight junction proteins and upregulated caveolin-1 and caveolae-mediated transcytotic vesicles [176,181,182]. BTB modulation by strengthening the activation of *K*_ATP_ can be tightly controlled by inhibitors and has been used via minoxidil to increase Herceptin delivery to primary or metastatic brain tumors [183,184,185,186]. Although minoxidil was found to be nontoxic in both mice and rats [175], nonselective activation of *K*_ATP_ may induce pericardial effusion, cardiac tamponade, reflex tachycardia, myocardial necrosis, coronary arteriopathy, degeneration of renal tubules, hypotension, dermatologic reactions, and hypertrichosis [154,187]. Intracarotid injection rather intravenous infusion holds the potential of concentrating minoxidil to the brain and reducing effects on peripheral tissues. As an alternative approach, in our previous work, minoxidil was delivered by hyaluronic acid modified nanoparticles to specially intensify the activation of BTB *K*_ATP_ to enhance specific accumulation of subsequently injected therapeutic nanoparticles in brain metastases in mice [188].

### 5.4. Other Potential Multifunctional Strategies

As a key factor in hypertension, diabetes and aging, angiotensin-II can increase BBB permeability in both paracellular and transcellular manner by altering the distribution of tight junction proteins, decreasing Mfsd2a, and increasing caveolin-1 [189]. Thus, angiotensin-II can be used to open the BBB for increased drug delivery into the brain to treat various brain diseases. As a surgical technique, laser interstitial thermal therapy has been widely used to ablate brain tumors [190,191]. Interestingly, increasing data indicate that thermal therapy can disrupt the BBB via temporarily altering tight junctions and increasing transcytosis [190]. Although this technique is invasive and requires general anesthesia, combination of laser interstitial thermal therapy with other systemic therapies still holds the potential for synergistic therapeutic effect.

## 6. Conclusions and Future Perspectives

Modulation of the BBB, including tight junctions, active efflux transporters, and transcytotic vesicles, has been extensively studied to increase drug delivery to the brain. Although improved intracranial drug concentrations were often shown for almost all approaches, most of these studies were conducted preclinically and focused on brain tumors with very few exceptions on epilepsy. Side effects associated with these modulating strategies need to be carefully handled to extend these technologies to various brain diseases, including neurodegenerative diseases. First, although any delivery route can be used including intravenous, intracarotid or stereotactic administration, these BBB modulation approaches by themselves (e.g., radiation and various modulators) can be severely toxic. Second, besides the BBB’s protective roles, BBB modulations are likely to impair the intracranial physiological functions of related targets, e.g., normal physiological actions of bradykinin B2 receptor, S1PR1, Pgp, Mfsd2a, LRP1, GLUT1, A2A adenosine receptor, and *K*_ATP_. Third, increased drug concentrations in normal brain and peripheral tissues resulting from efflux inhibition or tight junction opening may worsen side effects of subsequent therapeutic drugs. Fourth, unwanted accumulation of endogenous neurotoxic blood components and xenobiotics in normal brain regions (even specific accumulation in diseased regions) may lead to severe neurological complications. Therefore, the modulation window of various modulation strategies should be carefully investigated for safe clinical translation, especially those multifunctional strategies that combine multiple BBB modulations.

## Figures and Tables

**Table 1:** BBB regulation strategies and related advantages and disadvantages.

BBB Modulation Targets	Strategies	Advantages	Disadvantages
Tight junctions	Osmotic disruption	Transient and reversible	Nonselective, uncontrolled flow, invasive, anesthesia, and side effects
	Radiation-mediated disruption	Disease-specific	Unclear mechanisms and acute, subacute, and chronic dose-dependent toxicity
	Activating bradykinin B2 receptor	Disease-specific, rapid and transient	Limited application to only brain tumor and peripheral side effects
	Direct interference	Transient and reversible	Peripheral side effects
Active efflux	Direct Inhibition	Transient and reversible…	Tolerability concerns of the inhibitor, and side effects to both brain and peripheral tissues
	Targeting regulatory pathways	Disease-specific	Slow and side effects
Transcytosis	Upregulation of LRP1	Drug-specific	Slow and possible LRP1-associated side effects
	Inhibition of Mfsd2a	Transient and reversible	Possible Mfsd2a-associated side effects
	Upregulation of GLUT1	Efficient	Fasting-associated poor compliance

## References

[1] Andreone B.J., Chow B.W., Tata A., Lacoste B., Ben-Zvi A., Bullock K., Deik A.A., Ginty D.D., Clish C.B., Gu C. (2017). Blood-brain barrier permeability is regulated by lipid transport-dependent suppression of caveolae-mediated transcytosis. Neuron.

[2] Wang J.Z., Xiao N., Zhang Y.Z., Zhao C.X., Guo X.H., Lu L.M. (2016). Mfsd2a-based pharmacological strategies for drug delivery across the blood-brain barrier. Pharmacol. Res..

[3] Knowland D., Arac A., Sekiguchi K.J., Hsu M., Lutz S.E., Perrino J., Steinberg G.K., Barres B.A., Nimmerjahn A., Agalliu D. (2014). Stepwise recruitment of transcellular and paracellular pathways underlies blood-brain barrier breakdown in stroke. Neuron.

[4] Ben-Zvi A., Lacoste B., Kur E., Andreone B.J., Mayshar Y., Yan H., Gu C. (2014). Mfsd2a is critical for the formation and function of the blood-brain barrier. Nature.

[5] Armulik A., Genove G., Mae M., Nisancioglu M.H., Wallgard E., Niaudet C., He L., Norlin J., Lindblom P., Strittmatter K. (2010). Pericytes regulate the blood-brain barrier. Nature.

[6] Daneman R., Zhou L., Kebede A.A., Barres B.A. (2010). Pericytes are required for blood-brain barrier integrity during embryogenesis. Nature.

[7] Arvanitis C.D., Ferraro G.B., Jain R.K. (2020). The blood-brain barrier and blood-tumour barrier in brain tumours and metastases. Nat. Rev. Cancer.

[8] Han L., Jiang C. (2021). Evolution of blood-brain barrier in brain diseases and related systemic nanoscale brain-targeting drug delivery strategies. Acta Pharm. Sin. B.

[9] Abbott N.J., Ronnback L., Hansson E. (2006). Astrocyte-endothelial interactions at the blood-brain barrier. Nat. Rev. Neurosci..

[10] Gao X., Yue Q., Liu Y., Fan D., Fan K., Li S., Qian J., Han L., Fang F., Xu F. (2018). Image-guided chemotherapy with specifically tuned blood brain barrier permeability in glioma margins. Theranostics.

[11] Liu J., He Y., Zhang J., Li J., Yu X., Cao Z., Meng F., Zhao Y., Wu X., Shen T. (2016). Functionalized nanocarrier combined seizure-specific vector with p-glycoprotein modulation property for antiepileptic drug delivery. Biomaterials.

[12] Soffietti R., Ahluwalia M., Lin N., Ruda R. (2020). Management of brain metastases according to molecular subtypes. Nat. Rev. Neurol..

[13] Luo H., Shusta E.V. (2020). Blood-brain barrier modulation to improve glioma drug delivery. Pharmaceutics.

[14] Lochhead J.J., Yang J., Ronaldson P.T., Davis T.P. (2020). Structure, function, and regulation of the blood-brain barrier tight junction in central nervous system disorders. Front. Physiol..

[15] Pandit R., Chen L., Gotz J. (2020). The blood-brain barrier: Physiology and strategies for drug delivery. Adv. Drug Deliv. Rev..

[16] Bell R.D., Winkler E.A., Sagare A.P., Singh I., LaRue B., Deane R., Zlokovic B.V. (2010). Pericytes control key neurovascular functions and neuronal phenotype in the adult brain and during brain aging. Neuron.

[17] Yanagida K., Liu C.H., Faraco G., Galvani S., Smith H.K., Burg N., Anrather J., Sanchez T., Iadecola C., Hla T. (2017). Size-selective opening of the blood-brain barrier by targeting endothelial sphingosine 1-phosphate receptor 1. Proc. Natl. Acad. Sci. USA.

[18] Kodack D.P., Askoxylakis V., Ferraro G.B., Fukumura D., Jain R.K. (2015). Emerging strategies for treating brain metastases from breast cancer. Cancer Cell.

[19] Haluska M., Anthony M.L. (2004). Osmotic blood-brain barrier modification for the treatment of malignant brain tumors. Clin. J. Oncol. Nurs..

[20] Kroll R.A., Neuwelt E.A. (1998). Outwitting the blood-brain barrier for therapeutic purposes: Osmotic opening and other means. Neurosurgery.

[21] Siegal T., Rubinstein R., Bokstein F., Schwartz A., Lossos A., Shalom E., Chisin R., Gomori J.M. (2000). In vivo assessment of the window of barrier opening after osmotic blood-brain barrier disruption in humans. J. Neurosurg..

[22] van Vliet E.A., da Costa Araujo S., Redeker S., van Schaik R., Aronica E., Gorter J.A. (2007). Blood-brain barrier leakage may lead to progression of temporal lobe epilepsy. Brain J. Neurol..

[23] Marchi N., Angelov L., Masaryk T., Fazio V., Granata T., Hernandez N., Hallene K., Diglaw T., Franic L., Najm I. (2007). Seizure-promoting effect of blood-brain barrier disruption. Epilepsia.

[24] Ikeda M., Bhattacharjee A.K., Kondoh T., Nagashima T., Tamaki N. (2002). Synergistic effect of cold mannitol and na(+)/ca(2+) exchange blocker on blood-brain barrier opening. Biochem. Biophys. Res. Commun..

[25] Stapleton S., Jaffray D., Milosevic M. (2017). Radiation effects on the tumor microenvironment: Implications for nanomedicine delivery. Adv. Drug Deliv. Rev..

[26] Brown W.R., Thore C.R., Moody D.M., Robbins M.E., Wheeler K.T. (2005). Vascular damage after fractionated whole-brain irradiation in rats. Radiat. Res..

[27] van Vulpen M., Kal H.B., Taphoorn M.J., El-Sharouni S.Y. (2002). Changes in blood-brain barrier permeability induced by radiotherapy: Implications for timing of chemotherapy? (review). Oncol. Rep..

[28] Crowe W., Wang L., Zhang Z., Varagic J., Bourland J.D., Chan M.D., Habib A.A., Zhao D. (2019). Mri evaluation of the effects of whole brain radiotherapy on breast cancer brain metastasis. Int. J. Radiat. Biol..

[29] Teng F., Tsien C.I., Lawrence T.S., Cao Y. (2017). Blood-tumor barrier opening changes in brain metastases from pre to one-month post radiation therapy. Radiother. Oncol. J. Eur. Soc. Ther. Radiol. Oncol..

[30] Bouchet A., Potez M., Coquery N., Rome C., Lemasson B., Brauer-Krisch E., Remy C., Laissue J., Barbier E.L., Djonov V. (2017). Permeability of brain tumor vessels induced by uniform or spatially microfractionated synchrotron radiation therapies. Int. J. Radiat. Oncol. Biol. Phys..

[31] Lemasson B., Serduc R., Maisin C., Bouchet A., Coquery N., Robert P., Le Duc G., Tropres I., Remy C., Barbier E.L. (2010). Monitoring blood-brain barrier status in a rat model of glioma receiving therapy: Dual injection of low-molecular-weight and macromolecular mr contrast media. Radiology.

[32] Fang L., Sun X., Song Y., Zhang Y., Li F., Xu Y., Ma S., Lin N. (2015). Whole-brain radiation fails to boost intracerebral gefitinib concentration in patients with brain metastatic non-small cell lung cancer: A self-controlled, pilot study. Cancer Chemother. Pharmacol..

[33] Zeng Y.D., Liao H., Qin T., Zhang L., Wei W.D., Liang J.Z., Xu F., Dinglin X.X., Ma S.X., Chen L.K. (2015). Blood-brain barrier permeability of gefitinib in patients with brain metastases from non-small-cell lung cancer before and during whole brain radiation therapy. Oncotarget.

[34] Miller M.A., Chandra R., Cuccarese M.F., Pfirschke C., Engblom C., Stapleton S., Adhikary U., Kohler R.H., Mohan J.F., Pittet M.J. (2017). Radiation therapy primes tumors for nanotherapeutic delivery via macrophage-mediated vascular bursts. Sci. Transl. Med..

[35] Le Pechoux C., Laplanche A., Faivre-Finn C., Ciuleanu T., Wanders R., Lerouge D., Keus R., Hatton M., Videtic G.M., Senan S. (2011). Clinical neurological outcome and quality of life among patients with limited small-cell cancer treated with two different doses of prophylactic cranial irradiation in the intergroup phase iii trial (pci99-01, eortc 22003-08004, rtog 0212 and ifct 99-01). Ann. Oncol. Off. J. Eur. Soc. Med Oncol..

[36] Dietrich J., Monje M., Wefel J., Meyers C. (2008). Clinical patterns and biological correlates of cognitive dysfunction associated with cancer therapy. Oncologist.

[37] Emerich D.F., Dean R.L., Osborn C., Bartus R.T. (2001). The development of the bradykinin agonist labradimil as a means to increase the permeability of the blood-brain barrier: From concept to clinical evaluation. Clin. Pharmacokinet..

[38] de Vries N.A., Beijnen J.H., Boogerd W., van Tellingen O. (2006). Blood-brain barrier and chemotherapeutic treatment of brain tumors. Expert Rev. Neurother..

[39] Bartus R.T., Elliott P.J., Dean R.L., Hayward N.J., Nagle T.L., Huff M.R., Snodgrass P.A., Blunt D.G. (1996). Controlled modulation of bbb permeability using the bradykinin agonist, rmp-7. Exp. Neurol..

[40] Sanovich E., Bartus R.T., Friden P.M., Dean R.L., Le H.Q., Brightman M.W. (1995). Pathway across blood-brain barrier opened by the bradykinin agonist, rmp-7. Brain Res..

[41] Matsukado K., Inamura T., Nakano S., Fukui M., Bartus R.T., Black K.L. (1996). Enhanced tumor uptake of carboplatin and survival in glioma-bearing rats by intracarotid infusion of bradykinin analog, rmp-7. Neurosurgery.

[42] Borlongan C.V., Emerich D.F. (2003). Facilitation of drug entry into the cns via transient permeation of blood brain barrier: Laboratory and preliminary clinical evidence from bradykinin receptor agonist, cereport. Brain Res. Bull..

[43] Ford J., Osborn C., Barton T., Bleehen N.M. (1998). A phase i study of intravenous rmp-7 with carboplatin in patients with progression of malignant glioma. Eur. J. Cancer.

[44] Warren K., Jakacki R., Widemann B., Aikin A., Libucha M., Packer R., Vezina G., Reaman G., Shaw D., Krailo M. (2006). Phase ii trial of intravenous lobradimil and carboplatin in childhood brain tumors: A report from the children’s oncology group. Cancer Chemother. Pharmacol..

[45] Prados M.D., Schold S.C., Fine H.A., Jaeckle K., Hochberg F., Mechtler L., Fetell M.R., Phuphanich S., Feun L., Janus T.J. (2003). A randomized, double-blind, placebo-controlled, phase 2 study of rmp-7 in combination with carboplatin administered intravenously for the treatment of recurrent malignant glioma. Neuro Oncol..

[46] Deeken J.F., Loscher W. (2007). The blood-brain barrier and cancer: Transporters, treatment, and trojan horses. Clin. Cancer Res. Off. J. Am. Assoc. Cancer Res..

[47] Riley M.G., Kim N.N., Watson V.E., Gobin Y.P., LeBel C.P., Black K.L., Bartus R.T. (1998). Intra-arterial administration of carboplatin and the blood brain barrier permeabilizing agent, rmp-7: A toxicologic evaluation in swine. J. Neurooncol..

[48] Hashizume K., Black K.L. (2002). Increased endothelial vesicular transport correlates with increased blood-tumor barrier permeability induced by bradykinin and leukotriene c4. J. Neuropathol. Exp. Neurol..

[49] Rozhkova E.A. (2011). Nanoscale materials for tackling brain cancer: Recent progress and outlook. Adv. Mater..

[50] Haseloff R.F., Dithmer S., Winkler L., Wolburg H., Blasig I.E. (2015). Transmembrane proteins of the tight junctions at the blood-brain barrier: Structural and functional aspects. Semin. Cell Dev. Biol..

[51] Zihni C., Mills C., Matter K., Balda M.S. (2016). Tight junctions: From simple barriers to multifunctional molecular gates. Nat. Rev. Mol. Cell Biol..

[52] Nitta T., Hata M., Gotoh S., Seo Y., Sasaki H., Hashimoto N., Furuse M., Tsukita S. (2003). Size-selective loosening of the blood-brain barrier in claudin-5-deficient mice. J. Cell Biol..

[53] Campbell M., Kiang A.S., Kenna P.F., Kerskens C., Blau C., O’Dwyer L., Tivnan A., Kelly J.A., Brankin B., Farrar G.J. (2008). Rnai-mediated reversible opening of the blood-brain barrier. J. Gene Med..

[54] Campbell M., Hanrahan F., Gobbo O.L., Kelly M.E., Kiang A.S., Humphries M.M., Nguyen A.T., Ozaki E., Keaney J., Blau C.W. (2012). Targeted suppression of claudin-5 decreases cerebral oedema and improves cognitive outcome following traumatic brain injury. Nat. Commun..

[55] Tachibana K., Hashimoto Y., Shirakura K., Okada Y., Hirayama R., Iwashita Y., Nishino I., Ago Y., Takeda H., Kuniyasu H. (2021). Safety and efficacy of an anti-claudin-5 monoclonal antibody to increase blood-brain barrier permeability for drug delivery to the brain in a non-human primate. J. Control. Release Off. J. Control. Release Soc..

[56] Hashimoto Y., Zhou W., Hamauchi K., Shirakura K., Doi T., Yagi K., Sawasaki T., Okada Y., Kondoh M., Takeda H. (2018). Engineered membrane protein antigens successfully induce antibodies against extracellular regions of claudin-5. Sci. Rep..

[57] Hashimoto Y., Shirakura K., Okada Y., Takeda H., Endo K., Tamura M., Watari A., Sadamura Y., Sawasaki T., Doi T. (2017). Claudin-5-binders enhance permeation of solutes across the blood-brain barrier in a mammalian model. J. Pharmacol. Exp. Ther..

[58] Dithmer S., Staat C., Muller C., Ku M.C., Pohlmann A., Niendorf T., Gehne N., Fallier-Becker P., Kittel A., Walter F.R. (2017). Claudin peptidomimetics modulate tissue barriers for enhanced drug delivery. Ann. N. Y. Acad. Sci..

[59] Krug S.M., Amasheh S., Richter J.F., Milatz S., Gunzel D., Westphal J.K., Huber O., Schulzke J.D., Fromm M. (2009). Tricellulin forms a barrier to macromolecules in tricellular tight junctions without affecting ion permeability. Mol. Biol. Cell.

[60] Zeniya S., Kuwahara H., Daizo K., Watari A., Kondoh M., Yoshida-Tanaka K., Kaburagi H., Asada K., Nagata T., Nagahama M. (2018). Angubindin-1 opens the blood-brain barrier in vivo for delivery of antisense oligonucleotide to the central nervous system. J. Control. Release Off. J. Control. Release Soc..

[61] Krug S.M., Hayaishi T., Iguchi D., Watari A., Takahashi A., Fromm M., Nagahama M., Takeda H., Okada Y., Sawasaki T. (2017). Angubindin-1, a novel paracellular absorption enhancer acting at the tricellular tight junction. J. Control. Release Off. J. Control. Release Soc..

[62] Spampinato S.F., Obermeier B., Cotleur A., Love A., Takeshita Y., Sano Y., Kanda T., Ransohoff R.M. (2015). Sphingosine 1 phosphate at the blood brain barrier: Can the modulation of s1p receptor 1 influence the response of endothelial cells and astrocytes to inflammatory stimuli?. PLoS ONE.

[63] Fan L., Yan H. (2016). Fty720 attenuates retinal inflammation and protects blood-retinal barrier in diabetic rats. Investig. Ophthalmol. Vis. Sci..

[64] Gril B., Paranjape A.N., Woditschka S., Hua E., Dolan E.L., Hanson J., Wu X., Kloc W., Izycka-Swieszewska E., Duchnowska R. (2018). Reactive astrocytic s1p3 signaling modulates the blood-tumor barrier in brain metastases. Nat. Commun..

[65] Dusaban S.S., Chun J., Rosen H., Purcell N.H., Brown J.H. (2017). Sphingosine 1-phosphate receptor 3 and rhoa signaling mediate inflammatory gene expression in astrocytes. J. Neuroinflammation.

[66] Sanna M.G., Vincent K.P., Repetto E., Nguyen N., Brown S.J., Abgaryan L., Riley S.W., Leaf N.B., Cahalan S.M., Kiosses W.B. (2016). Bitopic sphingosine 1-phosphate receptor 3 (s1p3) antagonist rescue from complete heart block: Pharmacological and genetic evidence for direct s1p3 regulation of mouse cardiac conduction. Mol. Pharmacol..

[67] Iannitti T., Palmieri B. (2010). An update on the therapeutic role of alkylglycerols. Mar. Drugs.

[68] Erdlenbruch B., Alipour M., Fricker G., Miller D.S., Kugler W., Eibl H., Lakomek M. (2003). Alkylglycerol opening of the blood-brain barrier to small and large fluorescence markers in normal and c6 glioma-bearing rats and isolated rat brain capillaries. Br. J. Pharmacol..

[69] Erdlenbruch B., Jendrossek V., Eibl H., Lakomek M. (2000). Transient and controllable opening of the blood-brain barrier to cytostatic and antibiotic agents by alkylglycerols in rats. Exp. Brain.

[70] Erdlenbruch B., Jendrossek V., Kugler W., Eibl H., Lakomek M. (2002). Increased delivery of erucylphosphocholine to c6 gliomas by chemical opening of the blood-brain barrier using intracarotid pentylglycerol in rats. Cancer Chemother. Pharmacol..

[71] Erdlenbruch B., Schinkhof C., Kugler W., Heinemann D.E., Herms J., Eibl H., Lakomek M. (2003). Intracarotid administration of short-chain alkylglycerols for increased delivery of methotrexate to the rat brain. Br. J. Pharmacol..

[72] Kim H.J., Pyeun Y.S., Kim Y.W., Cho B.M., Lee T.H., Moon T.Y., Suh K.T., Park B.R. (2005). A model for research on the blood-brain barrier disruption induced by unsaturated fatty acid emulsion. Investig. Radiol..

[73] Sztriha L., Betz A.L. (1991). Oleic acid reversibly opens the blood-brain barrier. Brain Res..

[74] Zhou W., Chen C., Shi Y., Wu Q., Gimple R.C., Fang X., Huang Z., Zhai K., Ke S.Q., Ping Y.F. (2017). Targeting glioma stem cell-derived pericytes disrupts the blood-tumor barrier and improves chemotherapeutic efficacy. Cell Stem Cell.

[75] Guerra D.A.P., Paiva A.E., Sena I.F.G., Azevedo P.O., Silva W.N., Mintz A., Birbrair A. (2018). Targeting glioblastoma-derived pericytes improves chemotherapeutic outcome. Angiogenesis.

[76] Rodriguez P.L., Jiang S., Fu Y., Avraham S., Avraham H.K. (2014). The proinflammatory peptide substance p promotes blood-brain barrier breaching by breast cancer cells through changes in microvascular endothelial cell tight junctions. Int. J. Cancer.

[77] Ferrero E., Zocchi M.R., Magni E., Panzeri M.C., Curnis F., Rugarli C., Ferrero M.E., Corti A. (2001). Roles of tumor necrosis factor p55 and p75 receptors in tnf-alpha-induced vascular permeability. Am. J. Physiol. Cell Physiol..

[78] Connell J.J., Chatain G., Cornelissen B., Vallis K.A., Hamilton A., Seymour L., Anthony D.C., Sibson N.R. (2013). Selective permeabilization of the blood-brain barrier at sites of metastasis. J. Natl. Cancer Inst..

[79] Avraham H.K., Jiang S., Fu Y., Nakshatri H., Ovadia H., Avraham S. (2014). Angiopoietin-2 mediates blood-brain barrier impairment and colonization of triple-negative breast cancer cells in brain. J. Pathol..

[80] Gurnik S., Devraj K., Macas J., Yamaji M., Starke J., Scholz A., Sommer K., Di Tacchio M., Vutukuri R., Beck H. (2016). Angiopoietin-2-induced blood-brain barrier compromise and increased stroke size are rescued by ve-ptp-dependent restoration of tie2 signaling. Acta Neuropathol..

[81] Loscher W., Gericke B. (2020). Novel intrinsic mechanisms of active drug extrusion at the blood-brain barrier: Potential targets for enhancing drug delivery to the brain?. Pharmaceutics.

[82] Potschka H. (2010). Targeting regulation of abc efflux transporters in brain diseases: A novel therapeutic approach. Pharmacol. Ther..

[83] Bauer B., Hartz A.M., Fricker G., Miller D.S. (2005). Modulation of p-glycoprotein transport function at the blood-brain barrier. Exp. Biol. Med..

[84] Fox E., Bates S.E. (2007). Tariquidar (xr9576): A p-glycoprotein drug efflux pump inhibitor. Expert Rev. Anticancer Ther..

[85] Bors L.A., Erd F. (2019). Overcoming the blood–brain barrier. Challenges and tricks for cns drug delivery. Sci. Pharm..

[86] Shan Y., Cen Y., Zhang Y., Tan R., Zhao J., Nie Z., Zhang J., Yu S. (2021). Effect of p-glycoprotein inhibition on the penetration of ceftriaxone across the blood-brain barrier. Neurochem. Res..

[87] Erdo F., Nagy I., Toth B., Bui A., Molnar E., Timar Z., Magnan R., Krajcsi P. (2017). Abcb1a (p-glycoprotein) limits brain exposure of the anticancer drug candidate seliciclib in vivo in adult mice. Brain Res. Bull..

[88] Carcaboso A.M., Elmeliegy M.A., Shen J., Juel S.J., Zhang Z.M., Calabrese C., Tracey L., Waters C.M., Stewart C.F. (2010). Tyrosine kinase inhibitor gefitinib enhances topotecan penetration of gliomas. Cancer Res..

[89] Choo E.F., Kurnik D., Muszkat M., Ohkubo T., Shay S.D., Higginbotham J.N., Glaeser H., Kim R.B., Wood A.J., Wilkinson G.R. (2006). Differential in vivo sensitivity to inhibition of p-glycoprotein located in lymphocytes, testes, and the blood-brain barrier. J. Pharmacol. Exp. Ther..

[90] Ekins S., Ecker G.F., Chiba P., Swaan P.W. (2007). Future directions for drug transporter modelling. Xenobiotica; Fate Foreign Compd. Biol. Syst..

[91] Harmsen S., Meijerman I., Beijnen J.H., Schellens J.H. (2007). The role of nuclear receptors in pharmacokinetic drug-drug interactions in oncology. Cancer Treat Rev..

[92] Zastre J.A., Chan G.N., Ronaldson P.T., Ramaswamy M., Couraud P.O., Romero I.A., Weksler B., Bendayan M., Bendayan R. (2009). Up-regulation of p-glycoprotein by hiv protease inhibitors in a human brain microvessel endothelial cell line. J. Neurosci. Res..

[93] Bauer B., Hartz A.M., Fricker G., Miller D.S. (2004). Pregnane x receptor up-regulation of p-glycoprotein expression and transport function at the blood-brain barrier. Mol. Pharmacol..

[94] Huang H., Wang H., Sinz M., Zoeckler M., Staudinger J., Redinbo M.R., Teotico D.G., Locker J., Kalpana G.V., Mani S. (2007). Inhibition of drug metabolism by blocking the activation of nuclear receptors by ketoconazole. Oncogene.

[95] Bankstahl J.P., Hoffmann K., Bethmann K., Loscher W. (2008). Glutamate is critically involved in seizure-induced overexpression of p-glycoprotein in the brain. Neuropharmacology.

[96] Loscher W. (1998). Pharmacology of glutamate receptor antagonists in the kindling model of epilepsy. Prog. Neurobiol..

[97] Bauer B., Hartz A.M., Pekcec A., Toellner K., Miller D.S., Potschka H. (2008). Seizure-induced up-regulation of p-glycoprotein at the blood-brain barrier through glutamate and cyclooxygenase-2 signaling. Mol. Pharmacol..

[98] Zibell G., Unkruer B., Pekcec A., Hartz A.M., Bauer B., Miller D.S., Potschka H. (2009). Prevention of seizure-induced up-regulation of endothelial p-glycoprotein by cox-2 inhibition. Neuropharmacology.

[99] van Vliet E.A., Zibell G., Pekcec A., Schlichtiger J., Edelbroek P.M., Holtman L., Aronica E., Gorter J.A., Potschka H. (2010). Cox-2 inhibition controls p-glycoprotein expression and promotes brain delivery of phenytoin in chronic epileptic rats. Neuropharmacology.

[100] Kulkarni S.K., Dhir A. (2009). Cyclooxygenase in epilepsy: From perception to application. Drugs Today.

[101] Pekcec A., Unkruer B., Schlichtiger J., Soerensen J., Hartz A.M., Bauer B., van Vliet E.A., Gorter J.A., Potschka H. (2009). Targeting prostaglandin e2 ep1 receptors prevents seizure-associated p-glycoprotein up-regulation. J. Pharmacol. Exp. Ther..

[102] Ahmad A.S., Ahmad M., de Brum-Fernandes A.J., Dore S. (2005). Prostaglandin ep4 receptor agonist protects against acute neurotoxicity. Brain Res..

[103] Bilak M., Wu L., Wang Q., Haughey N., Conant K., St Hillaire C., Andreasson K. (2004). Pge2 receptors rescue motor neurons in a model of amyotrophic lateral sclerosis. Ann. Neurol..

[104] McCullough L., Wu L., Haughey N., Liang X., Hand T., Wang Q., Breyer R.M., Andreasson K. (2004). Neuroprotective function of the pge2 ep2 receptor in cerebral ischemia. J. Neurosci. Off. J. Soc. Neurosci..

[105] Spudich A., Kilic E., Xing H., Kilic U., Rentsch K.M., Wunderli-Allenspach H., Bassetti C.L., Hermann D.M. (2006). Inhibition of multidrug resistance transporter-1 facilitates neuroprotective therapies after focal cerebral ischemia. Nat. Neurosci..

[106] Cirrito J.R., Deane R., Fagan A.M., Spinner M.L., Parsadanian M., Finn M.B., Jiang H., Prior J.L., Sagare A., Bales K.R. (2005). P-glycoprotein deficiency at the blood-brain barrier increases amyloid-beta deposition in an alzheimer disease mouse model. J. Clin. Investig..

[107] Vogelgesang S., Cascorbi I., Schroeder E., Pahnke J., Kroemer H.K., Siegmund W., Kunert-Keil C., Walker L.C., Warzok R.W. (2002). Deposition of alzheimer’s beta-amyloid is inversely correlated with p-glycoprotein expression in the brains of elderly non-demented humans. Pharmacogenetics.

[108] Deane R., Zlokovic B.V. (2007). Role of the blood-brain barrier in the pathogenesis of alzheimer’s disease. Curr. Alzheimer Res..

[109] Lee G., Bendayan R. (2004). Functional expression and localization of p-glycoprotein in the central nervous system: Relevance to the pathogenesis and treatment of neurological disorders. Pharm. Res..

[110] Lim J.C., Kania K.D., Wijesuriya H., Chawla S., Sethi J.K., Pulaski L., Romero I.A., Couraud P.O., Weksler B.B., Hladky S.B. (2008). Activation of beta-catenin signalling by gsk-3 inhibition increases p-glycoprotein expression in brain endothelial cells. J. Neurochem..

[111] Ju X., Chen H., Miao T., Ni J., Han L. (2021). Prodrug delivery using dual-targeting nanoparticles to treat breast cancer brain metastases. Mol. Pharm..

[112] Khan N.U., Ni J., Ju X., Miao T., Chen H., Han L. (2021). Escape from abluminal lrp1-mediated clearance for boosted nanoparticle brain delivery and brain metastasis treatment. Acta Pharm. Sin. B.

[113] Ni J., Miao T., Su M., Khan N.U., Ju X., Chen H., Liu F., Han L. (2021). Psma-targeted nanoparticles for specific penetration of blood-brain tumor barrier and combined therapy of brain metastases. J. Control. Release Off. J. Control. Release Soc..

[114] Guo Q., Zhu Q., Miao T., Tao J., Ju X., Sun Z., Li H., Xu G., Chen H., Han L. (2019). Lrp1-upregulated nanoparticles for efficiently conquering the blood-brain barrier and targetedly suppressing multifocal and infiltrative brain metastases. J. Control. Release Off. J. Control. Release Soc..

[115] Guo Q., Chang Z., Khan N.U., Miao T., Ju X., Feng H., Zhang L., Sun Z., Li H., Han L. (2018). Nanosizing noncrystalline and porous silica material-naturally occurring opal shale for systemic tumor targeting drug delivery. ACS Appl. Mater. Interfaces.

[116] Dong A., Han L., Shao Z., Fan P., Zhou X., Yuan H. (2019). Glaucoma drainage device coated with mitomycin c loaded opal shale microparticles to inhibit bleb fibrosis. ACS Appl. Mater. Interfaces.

[117] Ju X., Miao T., Chen H., Ni J., Han L. (2021). Overcoming mfsd2a-mediated low transcytosis to boost nanoparticle delivery to brain for chemotherapy of brain metastases. Adv. Healthc. Mater..

[118] Uchida Y., Ohtsuki S., Katsukura Y., Ikeda C., Suzuki T., Kamiie J., Terasaki T. (2011). Quantitative targeted absolute proteomics of human blood-brain barrier transporters and receptors. J. Neurochem..

[119] Nguyen L.N., Ma D., Shui G., Wong P., Cazenave-Gassiot A., Zhang X., Wenk M.R., Goh E.L., Silver D.L. (2014). Mfsd2a is a transporter for the essential omega-3 fatty acid docosahexaenoic acid. Nature.

[120] Bengmark S. (2013). Gut microbiota, immune development and function. Pharmacol. Res..

[121] Alakbarzade V., Hameed A., Quek D.Q., Chioza B.A., Baple E.L., Cazenave-Gassiot A., Nguyen L.N., Wenk M.R., Ahmad A.Q., Sreekantan-Nair A. (2015). A partially inactivating mutation in the sodium-dependent lysophosphatidylcholine transporter mfsd2a causes a non-lethal microcephaly syndrome. Nat. Genet..

[122] Zhao Z., Sagare A.P., Ma Q., Halliday M.R., Kong P., Kisler K., Winkler E.A., Ramanathan A., Kanekiyo T., Bu G. (2015). Central role for picalm in amyloid-beta blood-brain barrier transcytosis and clearance. Nat. Neurosci..

[123] Andras I.E., Eum S.Y., Huang W., Zhong Y., Hennig B., Toborek M. (2010). Hiv-1-induced amyloid beta accumulation in brain endothelial cells is attenuated by simvastatin. Mol. Cell. Neurosci..

[124] Zandl-Lang M., Fanaee-Danesh E., Sun Y., Albrecher N.M., Gali C.C., Cancar I., Kober A., Tam-Amersdorfer C., Stracke A., Storck S.M. (2018). Regulatory effects of simvastatin and apoj on app processing and amyloid-beta clearance in blood-brain barrier endothelial cells. Biochim. Biophys. Acta Mol. Cell Biol. Lipids.

[125] Zlokovic B.V., Yamada S., Holtzman D., Ghiso J., Frangione B. (2000). Clearance of amyloid beta-peptide from brain: Transport or metabolism?. Nat. Med..

[126] Tobert J.A. (1987). New developments in lipid-lowering therapy: The role of inhibitors of hydroxymethylglutaryl-coenzyme a reductase. Circulation.

[127] Sweeney M.D., Zhao Z., Montagne A., Nelson A.R., Zlokovic B.V. (2019). Blood-brain barrier: From physiology to disease and back. Physiol. Rev..

[128] Sweeney M.D., Sagare A.P., Zlokovic B.V. (2018). Blood-brain barrier breakdown in alzheimer disease and other neurodegenerative disorders. Nat. Rev. Neurol..

[129] Winkler E.A., Nishida Y., Sagare A.P., Rege S.V., Bell R.D., Perlmutter D., Sengillo J.D., Hillman S., Kong P., Nelson A.R. (2015). Glut1 reductions exacerbate alzheimer’s disease vasculo-neuronal dysfunction and degeneration. Nat. Neurosci..

[130] Zhao C., Ma J., Wang Z., Li H., Shen H., Li X., Chen G. (2020). Mfsd2a attenuates blood-brain barrier disruption after sub-arachnoid hemorrhage by inhibiting caveolae-mediated transcellular transport in rats. Transl. Stroke Res..

[131] Montagne A., Zhao Z., Zlokovic B.V. (2017). Alzheimer’s disease: A matter of blood-brain barrier dysfunction?. J. Exp. Med..

[132] Guemez-Gamboa A., Nguyen L.N., Yang H., Zaki M.S., Kara M., Ben-Omran T., Akizu N., Rosti R.O., Rosti B., Scott E. (2015). Inactivating mutations in mfsd2a, required for omega-3 fatty acid transport in brain, cause a lethal microcephaly syndrome. Nat. Genet..

[133] Patching S.G. (2017). Glucose transporters at the blood-brain barrier: Function, regulation and gateways for drug delivery. Mol. Neurobiol..

[134] Anraku Y., Kuwahara H., Fukusato Y., Mizoguchi A., Ishii T., Nitta K., Matsumoto Y., Toh K., Miyata K., Uchida S. (2017). Glycaemic control boosts glucosylated nanocarrier crossing the bbb into the brain. Nat. Commun..

[135] Arsiwala T.A., Sprowls S.A., Blethen K.E., Adkins C.E., Saralkar P.A., Fladeland R.A., Pentz W., Gabriele A., Kielkowski B., Mehta R.I. (2021). Ultrasound-mediated disruption of the blood tumor barrier for improved therapeutic delivery. Neoplasia.

[136] Meng Y., Suppiah S., Surendrakumar S., Bigioni L., Lipsman N. (2018). Low-intensity mr-guided focused ultrasound mediated disruption of the blood-brain barrier for intracranial metastatic diseases. Front. Oncol..

[137] Alonso A. (2015). Ultrasound-induced blood-brain barrier opening for drug delivery. Front. Neurol. Neurosci..

[138] Sheikov N., McDannold N., Sharma S., Hynynen K. (2008). Effect of focused ultrasound applied with an ultrasound contrast agent on the tight junctional integrity of the brain microvascular endothelium. Ultrasound Med. Biol..

[139] Sheikov N., McDannold N., Vykhodtseva N., Jolesz F., Hynynen K. (2004). Cellular mechanisms of the blood-brain barrier opening induced by ultrasound in presence of microbubbles. Ultrasound Med. Biol..

[140] Deng J., Huang Q., Wang F., Liu Y., Wang Z., Wang Z., Zhang Q., Lei B., Cheng Y. (2012). The role of caveolin-1 in blood-brain barrier disruption induced by focused ultrasound combined with microbubbles. J. Mol. Neurosci. MN.

[141] Aryal M., Fischer K., Gentile C., Gitto S., Zhang Y.Z., McDannold N. (2017). Effects on p-glycoprotein expression after blood-brain barrier disruption using focused ultrasound and microbubbles. PLoS ONE.

[142] Ahmed N., Gandhi D., Melhem E.R., Frenkel V. (2021). Mri guided focused ultrasound-mediated delivery of therapeutic cells to the brain: A review of the state-of-the-art methodology and future applications. Front. Neurol..

[143] Chen K.T., Lin Y.J., Chai W.Y., Lin C.J., Chen P.Y., Huang C.Y., Kuo J.S., Liu H.L., Wei K.C. (2020). Neuronavigation-guided focused ultrasound (navifus) for transcranial blood-brain barrier opening in recurrent glioblastoma patients: Clinical trial protocol. Ann. Transl. Med..

[144] Appelboom G., Detappe A., LoPresti M., Kunjachan S., Mitrasinovic S., Goldman S., Chang S.D., Tillement O. (2016). Stereotactic modulation of blood-brain barrier permeability to enhance drug delivery. Neuro. Oncol..

[145] McDannold N., Zhang Y., Supko J.G., Power C., Sun T., Vykhodtseva N., Golby A.J., Reardon D.A. (2020). Blood-brain barrier disruption and delivery of irinotecan in a rat model using a clinical transcranial mri-guided focused ultrasound system. Sci. Rep..

[146] Hersh D.S., Wadajkar A.S., Roberts N., Perez J.G., Connolly N.P., Frenkel V., Winkles J.A., Woodworth G.F., Kim A.J. (2016). Evolving drug delivery strategies to overcome the blood brain barrier. Curr. Pharm. Des..

[147] Lammers T., Koczera P., Fokong S., Gremse F., Ehling J., Vogt M., Pich A., Storm G., van Zandvoort M., Kiessling F. (2015). Theranostic uspio-loaded microbubbles for mediating and monitoring blood-brain barrier permeation. Adv. Funct. Mater..

[148] Huang H.Y., Liu H.L., Hsu P.H., Chiang C.S., Tsai C.H., Chi H.S., Chen S.Y., Chen Y.Y. (2015). A multitheragnostic nanobubble system to induce blood-brain barrier disruption with magnetically guided focused ultrasound. Adv. Mater..

[149] Song Z., Wang Z., Shen J., Xu S., Hu Z. (2017). Nerve growth factor delivery by ultrasound-mediated nanobubble destruction as a treatment for acute spinal cord injury in rats. Int. J. Nanomed..

[150] Kinoshita M., McDannold N., Jolesz F.A., Hynynen K. (2006). Noninvasive localized delivery of herceptin to the mouse brain by mri-guided focused ultrasound-induced blood-brain barrier disruption. Proc. Natl. Acad. Sci. USA.

[151] Choi J.J., Selert K., Gao Z., Samiotaki G., Baseri B., Konofagou E.E. (2011). Noninvasive and localized blood-brain barrier disruption using focused ultrasound can be achieved at short pulse lengths and low pulse repetition frequencies. J. Cereb. Blood Flow Metab..

[152] Baseri B., Choi J.J., Tung Y.S., Konofagou E.E. (2010). Multi-modality safety assessment of blood-brain barrier opening using focused ultrasound and definity microbubbles: A short-term study. Ultrasound Med. Biol..

[153] Hynynen K., McDannold N., Vykhodtseva N., Jolesz F.A. (2001). Noninvasive mr imaging-guided focal opening of the blood-brain barrier in rabbits. Radiology.

[154] Wala K., Szlasa W., Saczko J., Rudno-Rudzinska J., Kulbacka J. (2021). Modulation of blood-brain barrier permeability by activating adenosine a2 receptors in oncological treatment. Biomolecules.

[155] Sassaroli E., O’Neill B.E. (2014). Modulation of the interstitial fluid pressure by high intensity focused ultrasound as a way to alter local fluid and solute movement: Insights from a mathematical model. Phys. Med. Biol..

[156] Burgess A., Nhan T., Moffatt C., Klibanov A.L., Hynynen K. (2014). Analysis of focused ultrasound-induced blood-brain barrier permeability in a mouse model of alzheimer’s disease using two-photon microscopy. J. Control. Release Off. J. Control. Release Soc..

[157] McMahon D., Poon C., Hynynen K. (2019). Evaluating the safety profile of focused ultrasound and microbubble-mediated treatments to increase blood-brain barrier permeability. Expert Opin. Drug Deliv..

[158] Effendi W.I., Nagano T., Kobayashi K., Nishimura Y. (2020). Focusing on adenosine receptors as a potential targeted therapy in human diseases. Cells.

[159] Ledent C., Vaugeois J.M., Schiffmann S.N., Pedrazzini T., El Yacoubi M., Vanderhaeghen J.J., Costentin J., Heath J.K., Vassart G., Parmentier M. (1997). Aggressiveness, hypoalgesia and high blood pressure in mice lacking the adenosine a2a receptor. Nature.

[160] Bynoe M.S., Viret C., Yan A., Kim D.G. (2015). Adenosine receptor signaling: A key to opening the blood-brain door. Fluids Barriers CNS.

[161] Bobermin L.D., Roppa R.H.A., Quincozes-Santos A. (2019). Adenosine receptors as a new target for resveratrol-mediated glioprotection. Biochim. Biophys. Acta Mol. Basis Dis..

[162] Kim D.G., Bynoe M.S. (2016). A2a adenosine receptor modulates drug efflux transporter p-glycoprotein at the blood-brain barrier. J. Clin. Investig..

[163] Jackson S., Anders N.M., Mangraviti A., Wanjiku T.M., Sankey E.W., Liu A., Brem H., Tyler B., Rudek M.A., Grossman S.A. (2016). The effect of regadenoson-induced transient disruption of the blood-brain barrier on temozolomide delivery to normal rat brain. J. Neurooncol..

[164] Vezina A., Manglani M., Morris D., Foster B., McCord M., Song H., Zhang M., Davis D., Zhang W., Bills J. (2021). Adenosine a2a receptor activation enhances blood-tumor barrier permeability in a rodent glioma model. Mol. cancer Res. MCR.

[165] Kim D.G., Bynoe M.S. (2015). A2a adenosine receptor regulates the human blood-brain barrier permeability. Mol. Neurobiol..

[166] Carman A.J., Mills J.H., Krenz A., Kim D.G., Bynoe M.S. (2011). Adenosine receptor signaling modulates permeability of the blood-brain barrier. J. Neurosci. Off. J. Soc. Neurosci..

[167] Pak R.W., Kang J., Valentine H., Loew L.M., Thorek D.L.J., Boctor E.M., Wong D.F., Kang J.U. (2018). Voltage-sensitive dye delivery through the blood brain barrier using adenosine receptor agonist regadenoson. Biomed. Opt. Express.

[168] Jackson S., Weingart J., Nduom E.K., Harfi T.T., George R.T., McAreavey D., Ye X., Anders N.M., Peer C., Figg W.D. (2018). The effect of an adenosine a2a agonist on intra-tumoral concentrations of temozolomide in patients with recurrent glioblastoma. Fluids Barriers CNS.

[169] Jackson S., George R.T., Lodge M.A., Piotrowski A., Wahl R.L., Gujar S.K., Grossman S.A. (2017). The effect of regadenoson on the integrity of the human blood-brain barrier, a pilot study. J. Neurooncol..

[170] Meng L., Wang C., Lu Y., Sheng G., Yang L., Wu Z., Xu H., Han C., Lu Y., Han F. (2021). Targeted regulation of blood-brain barrier for enhanced therapeutic efficiency of hypoxia-modifier nanoparticles and immune checkpoint blockade antibodies for glioblastoma. ACS Appl. Mater. Interfaces.

[171] Han L., Cai Q., Tian D., Kong D.K., Gou X., Chen Z., Strittmatter S.M., Wang Z., Sheth K.N., Zhou J. (2016). Targeted drug delivery to ischemic stroke via chlorotoxin-anchored, lexiscan-loaded nanoparticles. Nanomedicine.

[172] Han L., Kong D.K., Zheng M.Q., Murikinati S., Ma C., Yuan P., Li L., Tian D., Cai Q., Ye C. (2016). Increased nanoparticle delivery to brain tumors by autocatalytic priming for improved treatment and imaging. ACS Nano.

[173] Zou Y., Liu Y., Yang Z., Zhang D., Lu Y., Zheng M., Xue X., Geng J., Chung R., Shi B. (2018). Effective and targeted human orthotopic glioblastoma xenograft therapy via a multifunctional biomimetic nanomedicine. Adv. Mater..

[174] Gao X., Qian J., Zheng S., Changyi Y., Zhang J., Ju S., Zhu J., Li C. (2014). Overcoming the blood-brain barrier for delivering drugs into the brain by using adenosine receptor nanoagonist. ACS Nano.

[175] Khaitan D., Reddy P.L., Ningaraj N. (2018). Targeting brain tumors with nanomedicines: Overcoming blood brain barrier challenges. Curr. Clin. Pharmacol..

[176] Ningaraj N.S., Rao M.K., Black K.L. (2003). Adenosine 5′-triphosphate-sensitive potassium channel-mediated blood-brain tumor barrier permeability increase in a rat brain tumor model. Cancer Res..

[177] Brayden J.E. (2002). Functional roles of katp channels in vascular smooth muscle. Clin. Exp. Pharmacol. Physiol..

[178] Kitazono T., Faraci F.M., Taguchi H., Heistad D.D. (1995). Role of potassium channels in cerebral blood vessels. Stroke.

[179] Ruoslahti E. (2002). Specialization of tumour vasculature. Nat. Rev. Cancer.

[180] Ningaraj N.S., Sankpal U.T., Khaitan D., Meister E.A., Vats T.S. (2009). Modulation of kca channels increases anticancer drug delivery to brain tumors and prolongs survival in xenograft model. Cancer Biol. Ther..

[181] Gu Y.T., Xue Y.X., Wang Y.F., Wang J.H., Chen X., ShangGuan Q.R., Lian Y., Zhong L., Meng Y.N. (2013). Minoxidil sulfate induced the increase in blood-brain tumor barrier permeability through ros/rhoa/pi3k/pkb signaling pathway. Neuropharmacology.

[182] Gu Y.T., Xue Y.X., Zhang H., Li Y., Liang X.Y. (2011). Adenosine 5′-triphosphate-sensitive potassium channel activator induces the up-regulation of caveolin-1 expression in a rat brain tumor model. Cell. Mol. Neurobiol..

[183] Tinker A., Aziz Q., Thomas A. (2014). The role of atp-sensitive potassium channels in cellular function and protection in the cardiovascular system. Br. J. Pharmacol..

[184] Rich J.N., Bigner D.D. (2004). Development of novel targeted therapies in the treatment of malignant glioma. Nat. Rev. Drug Discov..

[185] Lockman P.R., Mittapalli R.K., Taskar K.S., Rudraraju V., Gril B., Bohn K.A., Adkins C.E., Roberts A., Thorsheim H.R., Gaasch J.A. (2010). Heterogeneous blood-tumor barrier permeability determines drug efficacy in experimental brain metastases of breast cancer. Clin. Cancer Res. Off. J. Am. Assoc. Cancer Res..

[186] Gallo J.M., Li S., Guo P., Reed K., Ma J. (2003). The effect of p-glycoprotein on paclitaxel brain and brain tumor distribution in mice. Cancer Res.

[187] Hanton G., Sobry C., Dagues N., Rochefort G.Y., Bonnet P., Eder V. (2008). Cardiovascular toxicity of minoxidil in the marmoset. Toxicol. Lett..

[188] Miao T.T., Ju X.F., Zhu Q.N., Wang Y.M., Guo Q., Sun T., Lu C.Z., Han L. (2019). Nanoparticles surmounting blood-brain tumor barrier through both transcellular and paracellular pathways to target brain metastases. Adv. Funct. Mater..

[189] Guo S., Som A.T., Arai K., Lo E.H. (2019). Effects of angiotensin-ii on brain endothelial cell permeability via pparalpha regulation of para- and trans-cellular pathways. Brain Res..

[190] Patel B., Yang P.H., Kim A.H. (2020). The effect of thermal therapy on the blood-brain barrier and blood-tumor barrier. Int. J. Hyperth..

[191] Ashraf O., Patel N.V., Hanft S., Danish S.F. (2018). Laser-induced thermal therapy in neuro-oncology: A review. World Neurosurg..

